# Cytoplasmic Lipid Membrane Biogenesis in Prokaryotes: From Soluble Lipid Building Blocks to a Dynamic and Functional Boundary Layer

**DOI:** 10.1002/cbic.70579

**Published:** 2026-10-23

**Authors:** Sergiy Gan, Marten Exterkate

**Affiliations:** ^1^ Membrane Biogenesis and Lipidomics Group Institute of Biochemistry Heinrich Heine University Düsseldorf Düsseldorf Germany

**Keywords:** asymmetric membranes, flip‐flop, lipid bilayer, lipid translocation, lysophosphatidic acid, membrane synthesis, phosphatidic acid

## Abstract

Prokaryotic membranes are fundamental to cellular organization and function, serving as dynamic platforms for transport, energy conservation, signaling, environmental adaptation, etc. This review provides an overview of prokaryotic membrane architecture, highlighting the structural diversity of bacterial and archaeal cell envelopes, focusing on the cytoplasmic membranes and the distinct lipid chemistries that define them. By describing the general pathways for fatty acid and phospholipid biosynthesis, we emphasize the universal role for the lipid intermediates lysophosphatidic acid and phosphatidic acid, as well as the major lipid species phosphatidylethanolamine, phosphatidylglycerol, and cardiolipin of the diderm bacteria *Escherichia coli*. Besides synthesis, lipid membrane insertion is described, highlighting recent findings regarding lipid leaflet distribution, asymmetry, and intermembrane transport. Altogether, these processes provide a general overview of lipid composition and function of proliferating cells.

## Graphical abstract

Lipid membranes are fundamental to all prokaryotic life. Besides acting as barrier, by orienting as hydrophobic bilayer, lipids are involved in a wide variety of other cellular processes. As a consequence, prokaryotic membranes are dynamic entities that switch their lipid composition as required; a process that requires tight interplay with the lipid synthesis machinery.

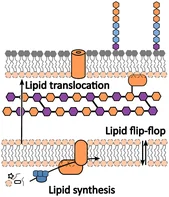

## 1 Prokaryotic Membrane Systems

Composed of substantial proportions of proteins and lipids [1], prokaryotic membranes function not only as physical permeability barriers but also as dynamic platforms that host a wide range of essential cellular processes, including metabolite transport, energy generation, signal transduction, and cell cycle regulation [2]. Rather than forming a uniform fluid mosaic [3], as previously assumed, prokaryotic membranes are now recognized as laterally organized structures containing distinct functional domains [4]. These membrane domains enable spatial segregation of biochemical activities without the need for internal membrane‐bound organelles and thus represent a fundamental organizational principle in prokaryotic cells [4].

In contrast to eukaryotes, prokaryotic organisms lack intracellular compartments defined by multiple membrane systems. Instead, the cytoplasmic membrane of prokaryotes is highly structured, dynamically regulated, and functionally specialized [5]. Prokaryotic membranes display remarkable structural and chemical diversity across phylogenetically distinct lineages, as well as within individual species that adapt to changing environmental conditions [6]. The cytoplasmic membrane consists of a (phospho)lipid bilayer that serves as a matrix in which membrane proteins are embedded. In bacteria, the lipids are predominantly composed of ester‐linked fatty acids. In contrast, archaeal membranes are typically built from ether‐linked isoprenoid lipids, which can assemble into either bilayer or monolayer architectures [6]. These fundamental chemical differences result in distinct biophysical properties, including variations in membrane fluidity, permeability, mechanical resilience, and thermal stability [7, 8]. Understanding these systems is essential for basic microbiology, as well as applied biomedical sciences, as membrane architecture influences a wide variety of processes, such as antibiotic susceptibility, environmental resilience, and pathogenicity [9, 10, 11]. In the following sections, the structural organization and functional characteristics of bacterial and archaeal membrane systems are described in more detail.

### 1.1 Bacterial Cell Envelopes

Historically, bacterial membrane systems are classified into two categories based on their reaction to Gram staining (Gram‐positive and Gram‐negative), a distinction that reflects fundamental differences in the cell envelope structure [12]. Although the Gram staining has been the gold standard for decades, there are an increasing number of exceptions reported. For example, mycobacteria contain an additional layer of mycolic acids (Mycomembrane) on top of the peptidoglycan layer, thereby hindering the Gram staining [13, 14]. Likewise, lineages branching at the root of *Bacillota* (formerly *Firmicutes*) phylum stain Gram‐negative, although they do not contain any known genes coding for outer membrane proteins or enzymes involved lipopolysaccharide biosynthesis [13]. To circumvent this, an updated nomenclature has been introduced: monoderm and diderm bacteria (Figure 1).

**FIGURE 1:**
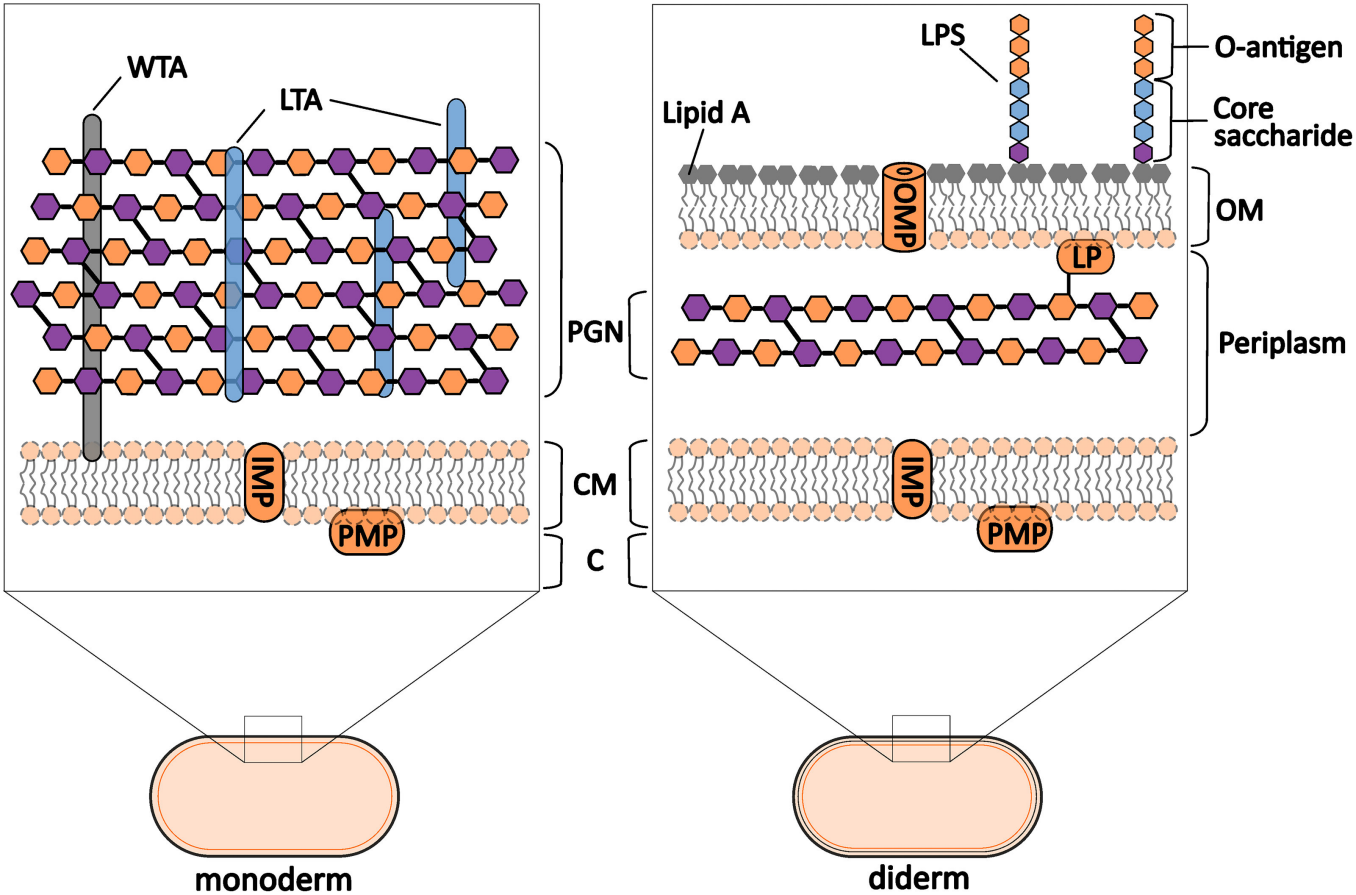
Schematic representation of the cell envelope of monoderm and diderm bacteria. Monoderm bacteria feature a single cytoplasmic membrane surrounded by a thick peptidoglycan (murein) cell wall (left panel). Diderm bacteria possess a double‐membrane system that consists of an inner membrane, a periplasm, and an outer membrane (right panel). Abbreviations: C, cytosol; CM, cytosolic membrane; IMP, integral membrane protein; PGN, peptidoglycan; PMP, peripheral membrane protein; OM, outer membrane; OMP, other membrane protein; LP, lipoprotein; LPS, lipopolysaccharide; LTA, lipoteichoic acid; WTA, wall teichoic acid.

### 1.2 Outer Envelope

Monoderm bacteria, previously referred to as Gram‐positive, possess a single cytoplasmic membrane commonly surrounded by a thick (40–80 nm) peptidoglycan (murein) cell wall. This multilayered elastic and net‐like peptidoglycan matrix contains proteins, as well as embedded teichoic and lipoteichoic acids, which contribute to surface charge and structural stability against certain environmental stressors. In addition, the outer surface of the monoderm cell envelope is enriched with a diverse array of proteins, which are either anchored via membrane‐spanning α‐helices or covalently linked to the peptidoglycan layer [15, 16]. In contrast, diderm bacteria, previously referred to as Gram‐negative, feature a complex double‐membrane envelope that consists of an inner (cytosolic) membrane, some form of an outer membrane, and commonly a small(er) peptidoglycan layer located in the periplasm. In contrast to monoderm bacteria, the peptidoglycan layer of diderm bacteria is considerably thinner, typically 7–8 nm [17]. This structural difference correlates with distinct mechanical properties of the cell envelope. Monoderm bacteria can withstand osmotic pressures of up to 20 atm, whereas diderm bacteria tolerate pressures of approximately 3 atm [18, 19]. Besides the peptidoglycan layer that serves as an additional permeability barrier, the periplasm contains varying (membrane) proteins involved in diverse cellular functions, including protein transport, folding, and oxidative processes [20]. The periplasm allows for the sequestration of proteins with potentially toxic effects in the cytoplasmic milieu and functions as a platform for important signaling and regulatory processes [20]. Notably, several ATP‐binding cassette (ABC) transporters and efflux pumps span both the inner and outer membranes, with essential components located within the periplasmic space [21].

Facing the extracellular environment, diderm bacteria possess the outer membrane, which constitutes the outermost permeability barrier of the cell envelope. The inner leaflet of the outer membrane is composed of phospholipids similar to those of the inner membrane, whereas the outer leaflet is enriched in lipopolysaccharides (LPS)–a glycolipid assembly that enhances barrier function and mediates interactions with host immune systems, reflecting a pronounced structural and compositional asymmetry between the two leaflets. LPS consists of lipid A, a nonrepeating core oligosaccharide, and a variable O‐antigen polysaccharide chain [22]. As an essential structural component of the outer membrane [23], LPS largely determines membrane permeability and confers resistance to numerous environmental toxins and clinically relevant antibiotics [24, 25].

### 1.3 Cytoplasmic Membrane

All components of the monoderm/diderm outer envelope are synthesized in the cytoplasm or at the cytoplasmic face of the inner membrane. For example, peptidoglycan precursors must be transported into the periplasm, where polymerization occurs, while all outer membrane components must traverse the aqueous periplasmic space to reach their final destination within the asymmetric outer membrane bilayer. As a consequence, all these components need to pass the cytoplasmic membrane. Indeed, the phospholipid cytoplasmic membrane, consisting of phospholipids, lipoproteins, and membrane proteins (integral and peripheral), is more than another hydrophobic barrier. Integral proteins of the inner membrane predominantly span the bilayer as α‐helical transmembrane segments enriched in hydrophobic residues [26]. Such α‐helical membrane proteins provide the conformational flexibility and dynamic rearrangements required for key cellular processes, including energy transduction, substrate transport, and signal transduction, within the phospholipid‐rich inner membrane environment [27, 28]. While eukaryotic cells have evolved complex intracellular organelles, separated by membrane boundaries to compartmentalize essential biochemical reactions, bacteria lack such internal membrane‐bound organelles. Instead, many functions that are compartmentalized in eukaryotes such as energy production, lipid biosynthesis, protein secretion, and transport processes are localized to, and coordinated at, the bacterial cytoplasmic membrane, in which the lipid matrix plays an active role.

Rather than representing a fixed lipid composition, prokaryotic cytoplasmic membranes exhibit substantial amount of lipid diversity, reflecting on physicochemical membrane requirements. The cytoplasmic membrane of diderm model organism *Escherichia coli* and its relatives comprises the common major lipid constituents phosphatidylethanolamine (PE), phosphatidylglycerol (PG), and smaller amounts of cardiolipin (CL), typically present in a zwitterionic:anionic molar ratio of approximately 70:30 [29]. Although these three lipid species are commonly present, numerous bacteria exist in which one of the species is missing. For example, the membrane of the model alphaproteobacterium *Caulobacter crescentus* consists of 87% PG and CL, whereas PE is not detected [30]. Besides that, the lipid composition of a certain organism is not fixed but can fluctuate due to various reasons, such as cell cycle stage, changing environmental conditions/stress, etc [31, 32]. During homeoviscous adaptation, bacteria modulate membrane fluidity and lipid packing by altering acyl chain length, degree of saturation, and branching in response to physicochemical stresses [33]. The different headgroup charges and molecular geometries of these lipids influence membrane properties, such as surface charge, curvature, and interactions with membrane‐associated proteins [34, 35]. In particular, the anionic lipid headgroups of PG and CL can contribute to electrostatic interactions with amphipathic helices of peripheral membrane proteins [34], whereas the zwitterionic PE supports membrane protein folding and topology [36]. Influenced by its headgroup and lipid tail sizes, lipids show a cylindrical or cone‐like shape on the three‐dimensional scale [35]. For example, CL, which has a conical molecular geometry, is often enriched in highly curved regions of bacterial membranes such as cell poles and division sites, thereby contributing to the negative inner membrane leaflet curvature orientation and spatial organization of membrane‐associated processes [37].

Beyond these three major phospholipid classes, bacteria can possess a wide variety of additional membrane lipids with diverse headgroups, which have been extensively reviewed by Sohlenkamp and Geiger [29]. For example, some diderm bacteria, including *Pseudomonas* species, synthesize lipids containing ornithine headgroups, which are polar membrane lipids that lack phosphate groups (Figure 4A) [39]. Under phosphate limitation, *Pseudomonas aeruginosa* produces ornithine lipids as primary membrane constituent to overcome phosphate‐limiting conditions [40]. The incorporation of such lipids illustrates that membrane composition is shaped not only by physicochemical requirements but also by the metabolic availability.

### 1.4 Archaeal Membrane Systems

Archaea constitute a distinct third domain of life that is evolutionarily and structurally separate from bacteria and eukaryotes. A defining feature of archaeal biology is the composition and organization of the cell envelope, which plays a central role in maintaining cellular integrity and adaptation, enabling survival across a wide range of ecological niches, including some of the most extreme environments [41]. Archaeal cell envelopes exhibit considerable structural diversity. In many archaeal species, the primary cell wall element is a surface layer (S‐layer) composed of self‐assembling protein or glycoprotein subunits arranged in a two‐dimensional crystalline lattice [6]. Its structure can take on several symmetries, such as oblique (p1 or p2), square (p4), or predominant hexagonal (p3 or p6) [42]. This S‐layer typically surrounds the cytoplasmic membrane and serves as the main protective and shape‐defining structure of the cell [42].

Certain archaeal groups, particularly some methanogens, possess an alternative polymer known as pseudomurein [43], which functionally resembles bacterial peptidoglycan but differs substantially in chemical composition. In contrast to bacterial peptidoglycan, which contains *N*‐acetylmuramic acid linked to D‐*N*‐acetylglucosamine (GlcNAc) via β‐1,4 glycosidic bonds, the carbohydrate backbone of pseudomurein is constructed from L‐*N*‐acetyltalosaminuronic acid connected to GlcNAc through β‐1,3 linkages [6, 43]. Moreover, the peptide cross‐links in pseudomurein differ fundamentally from those in bacterial cell walls as they do not incorporate D‐amino acids. Instead, the interpeptide bridge is commonly formed by a short chain of L‐amino acids, typically including glutamic acid, alanine, and lysine [6, 43, 44].

The cytoplasmic lipid membrane of archaea is chemically distinct from those of bacteria and eukaryotes. Archaeal lipids consist of isoprenoid chains typically linked to the glycerol‐phosphate backbone via ether bonds, a configuration that provides enhanced chemical stability compared to ester‐linked fatty acids. Furthermore, the glycerol backbone in archaeal lipids commonly displays opposite stereochemistry, which is considered as a distinct feature of evolutionary divergence between archaeal and bacterial membrane biosynthesis pathways [45]. It should be noted though that recently, several diderm bacteria belonging to the phylum bacillota (formerly known as Firmicutes) have been identified that contain lipids with the “archaeal” *sn*‐glycerol‐1‐phosphate lipid backbone, thereby questioning the strictness of the stereochemical segregation [46, 47, 48]. In many thermophilic and hyperthermophilic archaea, membrane lipids are further modified into tetraether structures that span the entire membrane, forming a continuous monolayer rather than a bilayer [49]. This configuration reduces membrane permeability and increases rigidity, contributing to membrane stability under extreme conditions, such as elevated temperatures, an acidic/alkaline environment, or high salinity [49, 50].

Importantly, archaeal cell envelopes are not built according to a uniform or universally conserved structural design. Rather, the wide range of envelope organizations, such as S‐layers, pseudomurein cell walls, and diverse lipid modifications, represents evolutionary adaptations that are specific to diverse ecological conditions. This architectural diversity enabled archaea to adjust envelope properties, including permeability, mechanical strength, and surface chemical characteristics, in response to environmental needs.

## 2 Phospholipid Biosynthesis Machinery

Among bacterial systems, *E. coli* has emerged as a benchmark model organism for the study of phospholipid biosynthesis [51]. Its well‐characterized lipid membrane architecture, extensive genetic tractability, and the availability of decades of biochemical and physiological data have enabled detailed dissection of lipid metabolic pathways. Many principles uncovered in *E. coli* have proven broadly applicable across bacteria, highlighting conserved strategies in phospholipid metabolism. At the same time, an increasing number of publications report deviations from the *E. coli* paradigm in other organisms, specifically regarding lipid headgroup biosynthesis, providing nuances for the domain of bacteria [5, 29].

In eukaryotes, lipid synthesis is distributed across multiple organelles, primarily the endoplasmic reticulum and mitochondria, and involves additional layers of regulation, compartmentalization, and lipid trafficking [52]. Despite these differences, several enzymatic reactions and lipid intermediates are evolutionarily conserved, allowing meaningful comparison with the bacterial system. In contrast, archaeal phospholipid biosynthesis diverges more fundamentally. Archaeal membranes are commonly composed of ether‐linked isoprenoid lipids; hence, their synthesis involves distinct enzymatic pathways, reflecting a deep evolutionary split from bacterial and eukaryotic lipid synthesis routes [45]. Comparing *E. coli* phospholipid biosynthesis with archaeal systems therefore provides insight into both conserved biochemical principles and alternative solutions to membrane construction.

### 2.1 Bacterial Lipid Tail Synthesis

#### 2.1.1 Fatty Acid Synthesis – Initiation

Fatty acid synthesis is an essential metabolic process that supplies the hydrophobic building blocks required for membrane phospholipids, lipoproteins, and signaling molecules. The initiation step (Figure 2A) of fatty acid biosynthesis is the formation of malonyl‐coenzyme A, generated from acetyl‐CoA by acetyl‐CoA carboxylase (AccABCD). Subsequently, the malonyl group is transferred to the acyl carrier protein (ACP) by malonyl‐CoA‐ACP transacylase (FabD), resulting in malonyl‐ACP. Interestingly, the low‐molecular‐weight protein ACP represents a major player in this pathway shielding hydrophobic fatty acid intermediates within its cavity while they are covalently attached to its 4′‐phosphopantetheine prosthetic group [53]. Additionally, ACP attachment is required for substrate recognition by the fatty acid synthase II (FAS II) enzymes [54]. The initial condensation reaction is catalyzed by β‐ketoacyl‐ACP synthase III (FabH), which combines malonyl‐ACP with a short‐chain acetyl‐CoA (C2–C5) primer to generate a β‐ketoacyl‐ACP intermediate [55]. This step commits carbon units to fatty acid biosynthesis and establishes the starting point for chain elongation [56]. Notably, FabH is bacterium‐specific and determines the nature of the primer substrate, giving rise to branched chain fatty acids in monoderm bacteria. In *E. coli*, FabH uses short acetyl‐CoA (C2) primer, while in *Mycobacterium tuberculosis,* it can utilize longer chain acyl‐CoA starters [57, 58].

**FIGURE 2:**
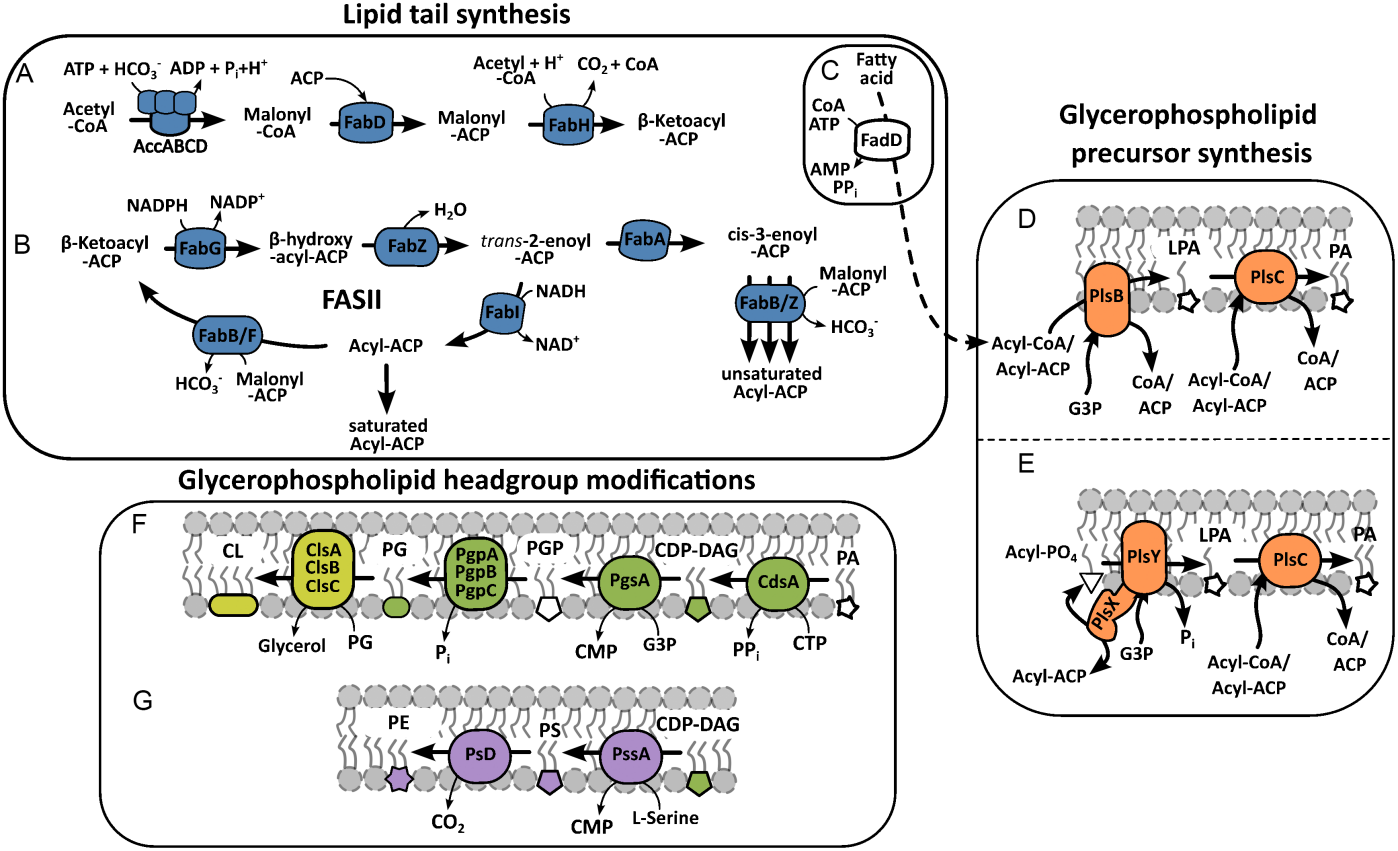
Schematic representation of glycerophospholipid synthesis in *Escherichia coli* divided into three sections: lipid tail synthesis (A–C), lipid tail attachment (D,E), and headgroup modifications (F,G). (A) Initiation of fatty acid synthesis with establishing carbon backbone from acetyl‐CoA and malonyl‐CoA for subsequent elongation. (B) Short, water‐soluble, carbon chains are assembled and elongated through multiple rounds of fatty acid synthesis, mediated by the type II fatty acid synthase (FAS II) system. One branch leads to the saturated fatty acid pool through continuous elongation by the FAS II machinery. The second branch diverges at FabA and leads to unsaturated fatty acid synthesis. The resulting unsaturated fatty acids are subsequently elongated by FabB/Z before being incorporated into membrane phospholipids. (C) Exogenous fatty acid uptake and activation by conjugation to coenzyme A, mediated by FadD. (D) Transfer of activated acyl chains to the glycerol‐phosphate backbone by the acyltransferases PlsB and PlsC result in the formation of the precursor PA. (E) Alternative route toward PA, via the PlsX/Y pathway: PlsX converts acyl‐ACP to acyl‐phosphate, PlsY transfers acyl‐phosphate to glycerol‐3‐phosphate to form LPA. (F) PA activation by CTP to form the central headgroup precursor CDP‐DAG. CDP‐DAG is converted to phosphatidylglycerol phosphate (PGP) via transfer of glycerol‐3‐phosphate, followed by dephosphorylation to yield phosphatidylglycerol (PG). Cardiolipin synthase (Cls) then transfers a phosphatidyl group from one PG molecule to another, producing cardiolipin (CL). (G) CDP‐DAG is converted to PS by substitution of CMP with L‐serine. PS is subsequently decarboxylated by phosphatidylserine decarboxylase (Psd) to generate PE.

#### 2.1.2 Fatty Acid Synthesis – Elongation

Fatty acid chain extension proceeds through a conserved, cyclic sequence of reactions (Figure 2A). Each elongation round adds two carbon atoms to the growing acyl chain and involves four enzymatic steps: condensation, reduction, dehydration, and a second reduction reaction [5]. This iterative mechanism is characteristic of the bacterial type II fatty acid synthase system, in which each catalytic activity is carried out by an individual enzyme rather than a multifunctional complex as found primary in eukaryotes, with exception of mycobacteria and corynebacteria [59]. Each elongation round begins with a Claisen condensation between a growing acyl group attached to ACP and a malonyl‐ACP molecule, catalyzed by β‐ketoacyl‐ACP synthases FabB and FabF [60]. Both enzymes have partially overlapping but distinct roles, particularly in the synthesis of unsaturated fatty acids and in determining final chain length distribution [61]. The decarboxylation of the malonyl moiety drives the reaction forward energetically, enabling formation of a new carbon–carbon bond [61]. As a result, the acyl chain is lengthened by two carbons, producing a β‐ketoacyl‐ACP intermediate. The newly formed β‐ketoacyl‐ACP undergoes stereospecific and NADPH‐dependent reduction of its keto group to a hydroxyl group. This reaction is catalyzed by the *E. coli* essential β‐ketoacyl‐ACP reductase (FabG) and results in the product β‐hydroxyacyl‐ACP (Figure 2B) [62, 63]. Next, β‐hydroxyacyl‐ACP was dehydrated to generate a trans‐2‐enoyl‐ACP intermediate. In *E. coli*, this reaction is catalyzed by either FabA or FabZ, depending on substrate specificity and physiological context [64]. FabZ functions primarily in saturated fatty acid elongation, whereas FabA (FabM in monoderm bacteria) plays a dual role by catalyzing both dehydration and isomerization reactions necessary for unsaturated fatty acid biosynthesis [64]. The final step of the elongation cycle is the reduction of the trans‐2‐enoyl‐ACP to acyl‐ACP. Using NADPH as an electron donor, this reaction is catalyzed by enoyl‐ACP reductase (FabI) [65]. With this reaction, the initial acyl‐ACP (C_*n*_) entering the cycle is extended by two carbons (C_*n*+2_). From here, the elongated acyl chain can either reenter the elongation cycle or move to the next step in the phospholipid biosynthesis pathway as a saturated acyl chain. Alternatively, FabA and FabB provide a route for unsaturated fatty acid synthesis in *E. coli*. This pathway proceeds through trans‐2‐decenoyl‐ACP, which is isomerized by FabA to cis‐3‐decenoyl‐ACP [64]. The resulting unsaturated intermediate is subsequently elongated by FabB before entering the phospholipid biosynthesis pathway.

As an alternative strategy, bacteria can incorporate exogenous fatty acids acquired from the environment [5, 66]. In *E. coli*, these fatty acids are activated by the acyl‐CoA synthetase (FadD), which converts them into acyl‐CoA thioesters that can serve as substrates for phospholipid biosynthesis (Figure 2C) [67]. This activation route has also been exploited as a shortcut in bottom‐up engineering approaches to synthetic cells [68]. However, once activated in this form, they cannot be further elongated by the type‐II fatty acid synthesis system. An alternative strategy involves β‐oxidation, a central catabolic pathway for fatty acids that feeds into the tricarboxylic acid (TCA) cycle to generate ATP by converting fatty acids into acetyl‐CoA [69, 70]. In the prokaryotic cytosol, CoA‐linked fatty acids undergo a four‐step cyclic process: (I) oxidation by acyl‐CoA dehydrogenase (FadE); (II) hydration by enoyl‐CoA hydratase (FadB); (III) a second oxidation by hydroxyacyl‐CoA dehydrogenase (FadB); and (IV) thiolysis by β‐ketothiolase (FadA) [71]. Each cycle yields one molecule of acetyl‐CoA and an acyl‐CoA shortened by two carbon atoms (C_*n*−2_) [71]. Although the main cellular function of β‐oxidation is energy generation, this pathway could be leveraged in an engineering approach to bypass the complex fatty acid synthesis FAS system by generating acetyl‐CoA or shorter acyl‐CoA intermediates that may be redirected into downstream lipid biosynthesis. Such concepts have also been discussed in the context of bottom‐up synthetic cell engineering, although full implementation of β‐oxidation in these systems has not yet been achieved.

#### 2.1.3 Regulation of Bacterial Fatty Acid Synthesis

In *E. coli*, palmitoyl‐ACP (C16:0), *cis*‐palmitoyl‐ACP (C16:1), and *cis*‐vaccenoyl‐ACP (C18:1) represent the major fatty acid products during standard exponential growth conditions [72]. Upon incorporation of these fatty acids as lipid tails of phospholipids, they form the core of the hydrophobic bilayer, in which the type and ratio of the fatty acid lipid tails largely determine membrane fluidity. This biophysical parameter directly impacts several fundamental properties of the membrane, such as integrity, permeability, and the propensity of membrane lipids to phase separate [73]. Membrane fluidity directly impacts the functioning of the embedded integral membrane proteins, local phase separation can contribute to the formation of microdomains, thereby supporting the needs of various membrane proteins [31]. This is a delicate balance, and alterations, such as lower membrane fluidity, can trigger unwanted lipid phase separation and protein segregation. As a consequence, essential cellular processes that involve the membrane, such as cytokinesis, envelope expansion, and maintenance of membrane potential, are hampered [74]. Focusing on the latter, membrane potential directly influences cellular respiration; a process that was shown to be tightly controlled by fluidizing lipids [75] Hence, it is of the utmost important that the optimal membrane fluidity is maintained and that upon changes in the environmental conditions, cells undergo homeoviscous adaption by triggering altered fatty acid synthesis. Indeed, the number of elongation cycles and thus the final chain length, degree of saturation, and branching position are influenced by environmental conditions, such as temperature [76]. At lower temperatures, the synthesis of unsaturated fatty acids is increased to maintain membrane fluidity [76]. Furthermore, the ratio of shorter‐chain to longer‐chain fatty acids rise at lower temperatures by exhibiting lower melting temperatures that compensate for decreased membrane fluidity. Conversely, at elevated temperatures, the proportion of saturated fatty acids rises, reducing membrane fluidity and preserving membrane stability [76]. Longer‐chain fatty acids enhance van der Waals interactions between lipid tails, therefore decreasing fluidity and increasing membrane rigidity [77]. Recently, it was discovered that in *E. coli,* the ratio between saturated and unsaturated fatty acid synthesis is regulated via the branchpoint enzymes FabI and FabB, thereby serving as a temperature‐sensitive switch [78]. This initial response is combined with a transcription‐based negative feedback loop to prevent overcompensation. Monoderm bacteria such as *Bacillus subtilis* further regulate membrane fluidity by adjusting the ratio of iso‐ and anteiso‐branched fatty acids [79]. At lower temperatures, *B. subtilis* increases the proportion of anteiso‐branched fatty acids, which carry a methyl branch at the penultimate carbon [79]. This lowers the melting temperature and enhances membrane fluidity [79]. In contrast, higher temperatures favor an increased proportion of iso‐branched fatty acids, which have higher melting temperatures and contribute to reduced membrane fluidity [79].

Furthermore, during growth phase transitions from exponential to stationary phase, *E. coli* alters its lipid composition as well. Specifically, *cis*‐palmitoleoyl‐ACP (C16:1) and *cis*‐vaccenoyl‐ACP (C18:1) are converted into the cyclopropane fatty acids, cyclopropane C17:0 (cyC17:0) and cyclopropane C19:0 (cyC19:0) upon entry into the stationary phase [80]. This modification is catalyzed by the enzyme cyclopropane fatty acid synthase, which adds a methylene group across the double bond of monounsaturated fatty acids [80]. The accumulation of cyclopropane fatty acids increases membrane rigidity and enhances resistance to environmental stresses such as oxidative stress and nutrient limitation, thereby contributing to cell survival [80, 81].

Additionally, fatty acid biosynthesis is tuned to membrane expansion during cell growth. Although expression of the *accABCD* operon, which encodes the subunits of acetyl‐CoA carboxylase that catalyze the first committed step of the pathway, scales with cellular growth rate [82], a proteomics study indicated that cellular ACC levels are not influenced, suggesting that the increase expression of the *accABCD* operon merely serves to maintain ACC levels [83]. Indeed, elevated transcription by plasmid‐encoded *accABCD* results in massive overproduction of its product malonyl‐CoA [84]. Only upon coupling with a thioesterase, an increase in fatty acid synthesis could be observed, indicating that not ACC but another component of the fatty acid synthesis pathway, that is, the levels of acyl‐ACP, regulate the flux [85, 86].

### 2.2 Archaeal Lipid Tail Synthesis

Besides the FAS II, some bacteria belonging to the CMN (Corynebacterium–Mycobacterium–Nocardia) group employ the type I fatty acid synthase (FAS I), which is also commonly utilized in Eukaryotes. In FAS I, all enzymatic activities required for fatty acid elongation are housed within a single, large, multifunctional protein complex (described in detail in the review article by Smith et al.) [87]. While the eukaryotic fatty acid synthesis process has some similarities with the bacterial, Archaea differ more markedly from *E. coli* in their lipid tail biosynthetic strategies. Although some archaeal species utilize fatty acid synthesis‐like reactions [88], most archaea do not rely on fatty acids as primary membrane components [45]. Instead, archaeal membranes are composed of isoprenoid chains synthesized via distinct metabolic routes [45]. These fundamental chemical differences are mirrored in the biosynthetic enzymes involved in lipid tail synthesis. Although archaeal lipid synthesis (extensively reviewed in [89, 90]) also starts with acetyl‐CoA, it utilizes the mevalonate (MVA) pathway to synthesize isopentenyl diphosphate (IPP) and dimethylallyl diphosphate (DMAPP). Subsequently, an elongation process is initiated, in which a growing isoprenoid chain is stepwise increased with 5 carbons each time, ultimately yielding geranylgeranyl diphosphate (GGPP). This process involves unique enzymes unrelated to bacterial FAS II components, highlighting that bacterial and archaeal systems utilize alternative evolutionary routes to membrane construction rather than variations on a single conserved pathway [45]. Nevertheless, despite these substantial differences in membrane building block synthesis, all three domains of life employ a similar principle: the stepwise elongation of water‐soluble precursors to build hydrophobic lipid tails, followed by their coupling to active carrier groups.

### 2.3 Synthesis of Central Bacterial Phospholipid Precursors

#### 2.3.1 PlsB/C Pathway

Phospholipid assembly begins with the formation of key early intermediates that establish the backbone and acyl chain framework of membrane lipids. In bacteria, lysophosphatidic acid (LPA) and phosphatidic acid (PA) are universal and essential intermediates that lead to the formation of all major bacterial glycero(phospho)lipids. In *E. coli*, the first committed step in glycerophospholipid synthesis is the acylation of G3P to form LPA. This reaction is catalyzed by glycerol‐3‐phosphate acyltransferase (PlsB), which ligates an acyl chain from an acyl‐ACP onto the *sn*‐1 position of G3P (Figure 2D). Subsequently, in the second step, the lysophosphatidic acid acyltransferase (PlsC) ligates an acyl chain from an acyl‐ACP to the *sn*‐2 position of LPA, yielding PA (Figure 2D). Because acyl‐ACP species are also intermediates of fatty acid biosynthesis, this step effectively couples early phospholipid synthesis with fatty acid metabolism. Both acyltransferases, PlsB and PlsC, are membrane‐attached (Figure 3A,C) and are able to not only utilize acyl‐ACP but also acyl‐CoA thioesters derived from exogenous fatty acids [66]. Structurally, PlsB and PlsC belong to the well‐characterized family of glycerolipid acyltransferases that share a conserved catalytic motif, typically represented as HX_4_D (Figure 3A,C) [92]. The histidine participates in deprotonation of glycerol’s hydroxyl group to facilitate nucleophilic attack on the thioster of the acyl donor, while the aspartate stabilizes the transition state and assists proton transfer [92].

**FIGURE 3:**
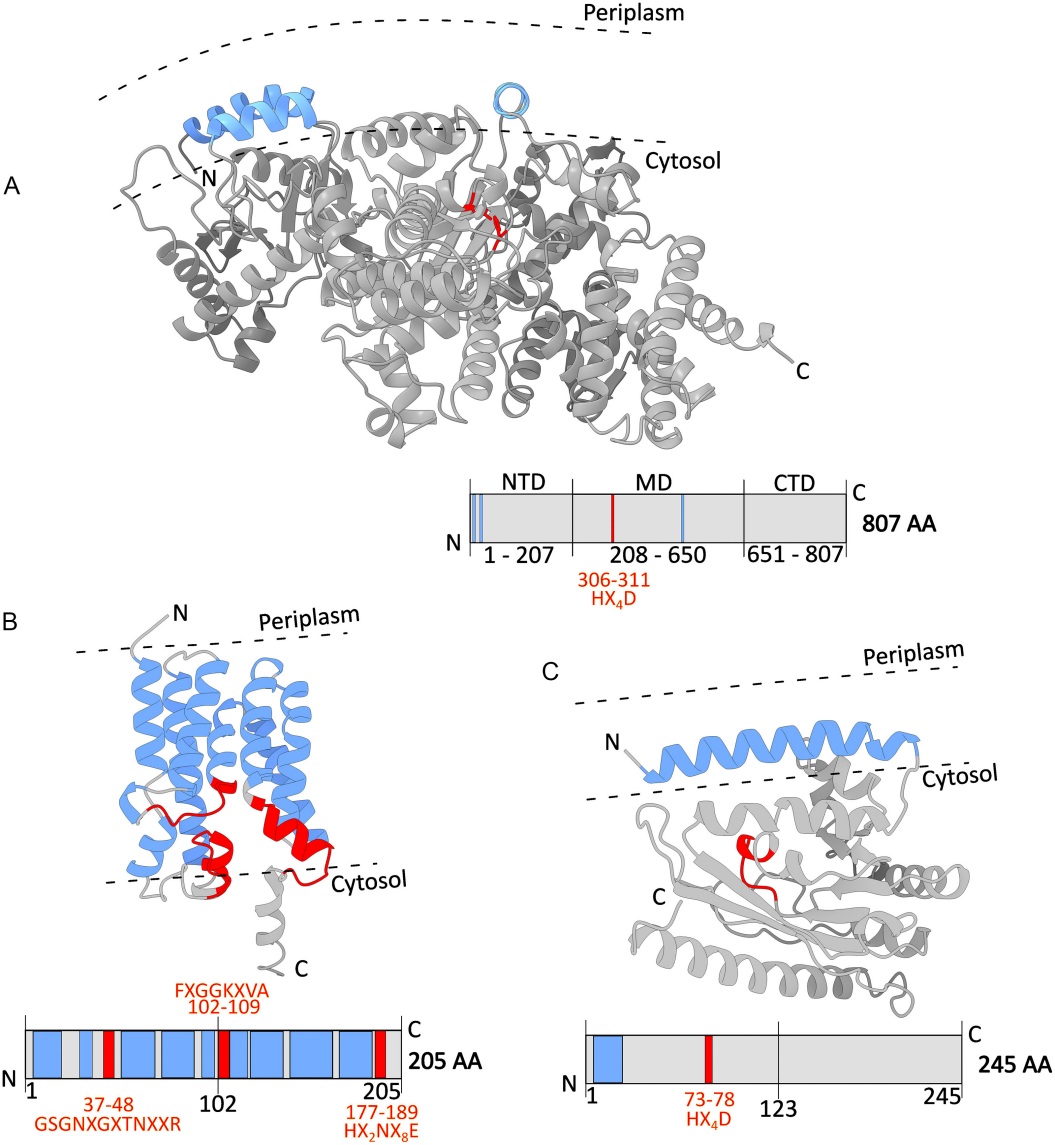
AlphaFold‐predicted model of PlsB, PlsY and PlsC**.** AlphaFold 3 model of PlsB (A), PlsY (B), and PlsC (C) with color‐coded structural features [91]. α‐Helices predicted to mediate interaction with inner membrane phospholipids are shown in blue, and parts contributing to protein activity are highlighted in red. The confidence rate of the predicted models can be found in the Figure S1 according to their per residue predicted local distance difference test scores (pLDDTs).

#### 2.3.2 PlsX/Y/C Pathway

Although present in *E. coli*, the PlsB/C pathway is not universally distributed throughout the domain of bacteria but predominantly associated with proteobacteria [66]. The alternative is formed by the PlsX/Y/C pathway, which is widely distributed among bacteria. This pathway converges with the canonical route at the level of phosphatidic acid (PA) formation but differs in both enzymatic organization and intermediate chemistry. As the glycerol‐3‐phosphate acyltransferase PlsY cannot directly utilize acyl‐ACP or acyl‐CoA as substrates, the enzyme PlsX first converts acyl‐ACP into acyl‐phosphate (Figure 2E) [66]. The resulting acyl‐phosphate intermediate is then used by the integral membrane protein PlsY (Figure 3B), which transfers the acyl group to the *sn*‐1 position of G3P, thereby generating LPA (Figure 2E) [93]. As described before, PlsC subsequently ligates an acyl chain from an acyl‐ACP to the *sn*‐2 position of LPA, yielding PA (Figure 2E). From a structural standpoint, PlsY lacks the canonical HX_4_D motif observed in PlsB and PlsC [66]. Its active site and catalytic residues are adapted to accommodate the chemically distinct acyl‐phosphate intermediate. Homology analyses comparing *E. coli* PlsY with the structurally characterized PlsY from *Streptococcus pneumoniae* identified three conserved motifs associated with catalytic activity (Figure 3B) [94]. Motif 1 spans amino acids 37–48 and comprises a glycine‐rich region containing a conserved arginine (R48), likely representing a phosphate‐binding loop commonly observed in nucleotide‐binding proteins [94]. Motif 2 extends from residues 102–109 and contains an essential lysine (K106), which is proposed to form a second phosphate‐binding site and contribute to substrate coordination [94]. Motif 3, encompassing amino acids 177–189, includes a conserved histidine (H177). This residue has been proposed to function analogously to the catalytic histidine in the PlsB HX_4_D motif, potentially acting as a general base that abstracts a proton from the hydroxyl group of the acyl acceptor during catalysis [94]. Functionally, the PlsX/Y combination decouples the initial acylation from FASII system through generating acyl‐PO_4_ as an intermediate for PlsY.

Although *E. coli*, as well as various other proteobacteria, contain both PlsB and PlsX/Y, it relies predominantly on the PlsB/C route for LPA formation and does not require PlsX/Y for phospholipid precursor synthesis [93]. Nevertheless, a double knockout of PlsX and PlsY results in synthetic lethality, although the precise physiological role of this pathway remains unclear. Within this pathway, PlsY is strictly dependent on PlsX activity as it requires acyl phosphate as a substrate for the acylation of G3P. An alternative role for *E*. *coli* PlsX has been hypothesized that is associated more with coordinating fatty acid and phospholipid metabolic flux than serving actively as part of a fully functional PlsX/Y pathway [93]. Likewise, PlsY is hypothesized to play an important role in maintaining LPA levels under conditions of metabolic imbalance or insufficient PlsB activity. However, these speculations require further research for validation. In contrast to proteobacteria, monoderm bacteria, as well as many other nonproteobacterial lineages, lack PlsB entirely and therefore depend exclusively on the PlsX/Y/C system for glycerophospholipid biosynthesis.

#### 2.3.3 Regulation of Key Enzymes in Early Phospholipid Biosynthesis

The synthesis of membrane phospholipids in *E. coli* is tightly coordinated with cellular growth, fatty acid production, and overall metabolic state. As established before, in the center of this process are the enzymes PlsB, PlsC, PlsX, and PlsY, involved in early‐stage phospholipid biosynthesis leading to catalysis and membrane insertion of the universal glycerophospholipid precursors LPA and PA. Rather than being regulated solely at the transcriptional level, these enzymes seem to be primarily controlled by substrate availability and through metabolic signals [5, 83, 93].

PlsB catalyzes the initial acylation of G3P and therefore represents the first committed step in phospholipid biosynthesis in *E. coli*. Its activity is closely linked to the cellular metabolic state. One important regulatory signal is the alarmone guanosine tetraphosphate (ppGpp), which accumulates during nutrient limitation and environmental stress. Elevated ppGpp levels influence the expression of numerous genes, including *plsB*, and have also been suggested to reduce PlsB activity at the posttranslational level, potentially through allosteric interactions [83]. As a consequence, its substrate acyl‐ACP will accumulate, which will have an inhibiting effect on fatty acid synthesis. Hence, PlsB plays a central role in regulating phospholipid synthesis. In contrast, PlsC appears to be regulated mainly by substrate availability rather than by direct allosteric control. Its activity depends on the supply of intermediates generated by PlsB and PlsY, linking it closely to fatty acid synthesis. This coupling ensures efficient conversion of acyl‐ACP intermediates into the key phospholipid precursor PA while minimizing the accumulation of LPA in the membrane [5].

### 2.4 Membrane Lipid Precursor Synthesis in Archaea

As previously mentioned, Archaea do not use fatty acids as their lipid tails. Instead, their membranes are composed of isoprenoid chains that are ether‐linked to a *sn‐*glycerol‐1‐phosphate (G1P) backbone [45, 89]. As a consequence, additional steps in archaeal lipid biosynthesis rely on enzymes that are not homologous to those found in bacterial or eukaryotic pathways. In Archaea, the enzymes geranylgeranyl glycerol‐phosphate synthase (GGGPS) and 2,3‐bis‐O‐geranylgeranyl‐sn‐glycerol‐1‐phosphate synthase (DGGGPS) are involved in the attachment of the isoprenoid lipid tails to the *sn‐*glycerol‐1‐phosphate lipid backbone (extensively reviewed by de Kok et al. [90]). Likewise, the early intermediates of this pathway, geranylgeranyl glycerol‐phosphate (GGGP) and 2,3‐bis‐O‐geranylgeranyl‐sn‐glycerol‐1‐phosphate (DGGGP), are chemically distinct from LPA and PA, although GGGP and LPA are both lysolipids, and one could consider DGGGP the archaeal equivalent of PA. Altogether, despite clear chemical differences, the archaeal pathway follows comparable principles for lipid tail attachment and membrane insertion: it generates hydrophobic building blocks and attaches them to a glycerol‐phosphate backbone, thereby also forming glycerophosphate lipid intermediates.

### 2.5 Bacterial Phospholipid Headgroup Modifications

In bacteria, phospholipid biosynthesis converges at phosphatidic acid (PA), which serves as the central precursor for all major membrane phospholipids. Beyond its metabolic role as a branching intermediate, PA also has important biophysical properties that influence membrane organization. Due to its relatively small, negatively charged phosphate headgroup combined with two bulky hydrophobic acyl chains, PA adopts a cone‐shaped molecular geometry [95, 96]. This geometry generates negative spontaneous curvature stress within the membrane lipid bilayers and favors the formation of nonbilayer structures such as inverted hexagonal phases or inverted micellar arrangements [95, 96]. Such structures are generally incompatible with stable bilayer organization. Bacteria can also synthesize non‐phosphate‐containing lipids with diverse headgroups and modifications, as reviewed by Sohlenkamp and Geiger [29]. However, this section focuses on the most abundant lipid species across the bacterial domain, namely, phosphatidylethanolamine (PE), phosphatidylglycerol (PG), and cardiolipin (CL), as exemplified by *E. coli*.

PE is the most abundant phospholipid in many bacteria and is characterized by a zwitterionic headgroup at physiological pH. The negatively charged phosphate group and the positively charged ethanolamine moiety result in an overall neutral molecule. Because its headgroup is relatively small compared to its hydrophobic acyl chains, PE adopts a cone‐shaped geometry [97]. This shape promotes negative membrane curvature and disfavors planar bilayer arrangements, similar to the behavior observed for PA. CL is structurally distinct (Figure 4B), consisting of two phosphatidyl units connected by a central glycerol backbone and carrying a highly anionic headgroup with an overall negative charge. Its relatively small headgroup combined with four acyl chains gives rise to a pronounced conical geometry in presence of divalent cations [98]. As a result, CL can strongly promote negative curvature, favoring nonlamellar membrane structures. CL tends to accumulate in regions of high curvature, such as cell poles and division sites [37, 98]. In contrast, PG has a more balanced headgroup‐to‐tail ratio, resulting in a cylindrical molecular shape [97]. This geometry supports the formation of stable lamellar bilayers and counteracts the curvature‐inducing effects of PE and CL [97]. In addition, PG is an anionic phospholipid with a net negative charge at physiological pH, contributing to the electrostatic properties of the membrane surface and facilitating interactions with positively charged ions and proteins [97, 99].

**FIGURE 4:**
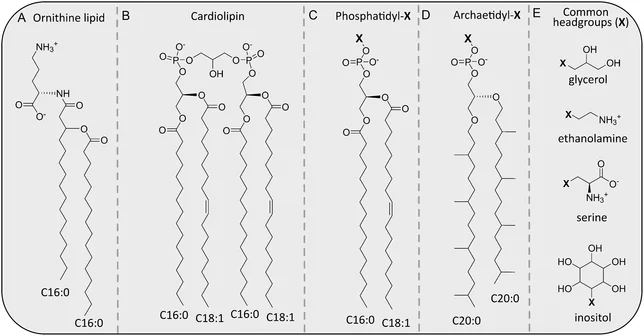
Structural diversity of bacterial and archaeal membrane lipids. (A) Proposed structure of phosphor‐free ornithine‐containing lipid linked to a backbone with palmitoyl (C16:0) moieties of *Pseudomonas* spp [38]. (B) Structural representation of the common cardiolipin with oleoyl (C18:1) palmitoyl (C16:0) moieties [29]. (C) The bacterial phospholipid backbone and (D) the archaeal phospholipid backbone. In both cases, the polar headgroup is represented by **X**, emphasizing the modularity membrane lipid architecture [29]. The phosphatidyl backbone is ester‐linked to fatty acyl chains, whereas the archaetidyl backbone is ether‐linked to isoprenoid chains. (E) Common bacterial and archaeal headgroups, including ethanolamine, glycerol, serine, and inositol [29].

#### 2.5.1 PE Synthesis

In *E. coli*, the first committed step in headgroup diversification is the activation of PA by CTP to produce CDP‐diacylglycerol (CDP‐DAG), catalyzed by CDP‐diacylglycerol synthase (CdsA) [100, 101]. CDP‐DAG is a key metabolic node from which the three main phospholipids of *E. coli* membranes are generated: PE, PG, and CL. PE constitutes roughly 75% of total membrane phospholipids during active growth. The formation of PE begins with phosphatidylserine synthase (PssA), which catalyzes an exchange reaction in which serine replaces cytidine monophosphate (CMP) on CDP‐DAG to yield phosphatidylserine (PS) (Figure 2G) [100, 102]. The PS intermediate is rapidly decarboxylated by phosphatidylserine decarboxylase (Psd), producing PE [100, 102]. PE is a zwitterionic phospholipid with an overall neutral charge, whereas PG carries a net negative charge due to its acidic headgroup (Figure 4C,E). The relative abundance of these two lipids establishes a dynamic balance of membrane surface charge, which is essential for proper folding, insertion, and topology of integral membrane proteins [103, 104]. PssA plays a critical role in this process as it requires acidic phospholipids for membrane association and activity [105]. This establishes a homeostatic feedback loop that maintains a stable ratio of PG to PE: low levels of PG and CL reduce PssA activity and consequently decrease PE synthesis, whereas higher levels of PG and CL enhance PssA activity and promote PE production [105].

#### 2.5.2 PG and CL Synthesis

PG and CL account for approximately 30% of *E. coli* membrane phospholipids, and their biosynthesis starts at CDP‐DAG. Phosphatidylglycerol‐phosphate synthase (PgsA) transfers G3P to CDP‐DAG to form phosphatidylglycerol phosphate (PGP) [102, 106]. This intermediate is subsequently dephosphorylated by one of several phosphatidylglycerol phosphate phosphatases (PgpA, PgpB, or PgpC) to produce PG (Figure 2F) [102, 106]. Under conditions of low osmolarity, *E. coli* synthesizes osmoregulated periplasmic glucans (OPGs) [107]. These periplasmic oligosaccharides are modified by the addition of phosphoglycerol groups derived directly from PG. This transfer reaction generates DAG as a byproduct [107]. DAG is subsequently recycled by the diacylglycerol kinase DgkA, which uses ATP to phosphorylate DAG to PA, producing ADP in the process [108]. This reaction prevents the accumulation of DAG within the membrane and replenishes phospholipid precursors for further headgroup synthesis. In this way, the pathway connects osmotic adaptation with membrane lipid homeostasis.

CL typically represents a smaller fraction of the total anionic lipid composition, although its abundance fluctuates based on various growth conditions [37]. In *E. coli,* cardiolipin is synthesized by cardiolipin synthases (ClsA, ClsB, and ClsC), which catalyze condensation reactions involving two PG molecules to form CL. It should be noted that cardiolipin is not essential for growing *E. coli* under specific laboratory conditions, as exemplified by a viable *clsA/B/C* triple knockout mutant [109]. Nevertheless, cardiolipin is associated with a wide variety of different processes, including environmental stress adaptation, illustrating its importance in natural growth [110, 111].

### 2.6 Lipid Diversity in Archaea

The enzymes that attach polar headgroups in archaea belong to related (bacterial) families of CDP‐alcohol phosphotransferases. Although they often show significant divergence in phospholipid substrate specificity [45, 90], some of the archaeal enzymes show remarkable activity toward bacterial phospholipid intermediate substrates and vice versa [112, 113]. Altogether, this reflects an evolutionary adaptation to archaeal membrane chemistry while at the same time maintaining bacterial substrate promiscuity. Noteworthy, significant numbers in archaeal species are able to deploy unique tetraether lipids, forming a continuous monolayer rather than a bilayer [49]. The ether linkages, isoprenoid chains, and bipolar tetraether lipids are significant for the physicochemical properties of archaeal lipid membranes, for example, their phase behavior and the permeability of the membranes [45, 114].

### 2.7 Lipid Transport in Prokaryotes

#### 2.7.1 Lipid Flip‐Flop

Flip‐flop, the translocation of lipids between two leaflets of one membrane bilayer, is an essential process during prokaryotic cellular growth. Novel lipids, synthesized on the inner leaflet of the cytosolic membrane, need to be transferred to the outer leaflet to maintain balanced expansion of both membrane leaflets. This process is energetically unfavorable due to the polar headgroups crossing the hydrophobic tail region. Depending on the type of lipid and concentration gradient, spontaneous flipflop occurs in a time scale from minutes to hours [115, 116]. Hence, to ensure lipid flipflop at physiologically relevant time scales, translocation needs to be aided. In this process, transmembrane peptides/proteins might play a facilitating role as they have been associated with increased lipid translocation rates [117, 118, 119]. As many of the tested membrane proteins have other primary function, and the lipid translocation is uncoupled and passive, this is currently considered to be a side effect. Lipid flip‐flop also plays a crucial role in in maintaining membrane asymmetry, a phenomenon that for long was thought to be exclusive for eukaryotes. However, recent literature showed that the inner membrane of *E. coli* is an asymmetric phospholipid bilayer in which lipids such as phosphatidylethanolamine (PE) are preferentially enriched in the cytoplasmic leaflet, resulting in dynamic, unequal distributions (e.g., ~75% vs. 25%) between the two leaflets [120]. To actively maintain such asymmetry, dedicated enzymes would be required [120]. Indeed, a recent study indicates that bacteria encode specific lipid transport systems. For example, the DedA family protein PetA promotes the transbilayer movement of PE in *B. subtilis*, suggesting that lipid flip‐flop can be actively mediated rather than relying solely on spontaneous diffusion [121]. Although in the diderm bacterium *E. coli*, no specific phospholipid flippase has yet been identified, it is expected that such putative lipid transport proteins also exist in this model organism. Altogether, lipid asymmetry is a dynamic, metabolically regulated process that contributes to differences in bilayer properties, such as lipid packing and order [120].

Lipid flip‐flop in archaea remains even less well understood. Although lipid redistribution is essential for membrane assembly and homeostasis, archaeal lipids differ significantly from bacterial lipids in both chemical structure and architecture as they can form either bilayer or monolayer membranes. In particular, tetraether lipids increase membrane rigidity and are likely to reduce spontaneous flip‐flop rates [49]. Most likely, specialized, yet still unidentified, transport mechanisms are required to facilitate lipid translocation in archaeal membranes as well.

#### 2.7.2 Lipid Translocation Between Inner and Outer Membrane

Diderm bacteria do not only require transport of newly synthesized lipids within the cytosolic membrane but also toward the outer membrane, which is characterized by a strong asymmetry with lipopolysaccharides enriched in the outer leaflet, whereas the inner leaflet is made up of phospholipids. Although the transport of LPS is extensively studied (see recent reviews [122, 123]), the transfer of phospholipids is only partially known. A key aspect is the preservation of the outer membrane asymmetry, which requires tightly controlled, multicomponent transport systems that shuttle lipids across the periplasm while preventing their accumulation in unwanted locations within the membrane. One key system involved in this process is the Mla pathway, which functions in the removal of mis‐localized phospholipids from the outer membrane and transports them back to the inner membrane [124, 125]. This retrograde transport is essential to prevent the buildup of phospholipids in the outer leaflet, which would otherwise disrupt membrane integrity and increase permeability [124].

In addition, a second class of proteins, known as AsmA‐like proteins, contributes to phospholipid transport across the cell envelope. Members of this family include YhdP, which is involved in maintaining outer membrane integrity and permeability, TamB, a component of the translocation and assembly module, and YdbH, which shares structural similarities with YhdP and TamB but whose precise function remains less well‐defined [126]. These proteins are proposed to form extended structures that span the periplasm and facilitate lipid movement between the inner and outer membranes [126, 127]. In contrast to the Mla system, which primarily supports retrograde transport, AsmA‐like proteins are thought to mediate anterograde lipid transport from the inner membrane to the outer membrane. Mechanistic models and structural predictions for this class of proteins have been discussed in detail by Kumar and Ruiz [128]. Together, the Mla pathway and AsmA‐like proteins represent complementary systems that maintain lipid homeostasis in diderm bacteria. While the Mla pathway retrieves phospholipids from the outer membrane to preserve lipid asymmetry, AsmA‐like proteins are proposed to support the delivery of phospholipids to the outer membrane.

## 3 Conclusion

Membrane biogenesis is a dynamic and highly coordinated process that extends beyond the synthesis of individual lipid molecules. Throughout this review, we have highlighted how the construction and maintenance of the cytoplasmic lipid membrane require the integration of metabolic pathways, enzymatic activities, membrane organization, and lipid transport. Fatty acid and phospholipid biosynthesis provide the molecular building blocks for membrane expansion, while the composition and physicochemical properties of these lipids determine fundamental membrane characteristics.

The bacterial systems discussed here, particularly those of the diderm *E. coli*, further illustrate that phospholipid synthesis is closely connected with cellular growth and membrane organization. The early steps of phospholipid biosynthesis converge on the formation of LPA and PA, linking fatty acid metabolism to membrane expansion. The activities of PlsB, PlsC, PlsX, and PlsY are therefore not simply components of isolated enzymatic pathways but represent points at which metabolic flux can be coordinated with the demand for membrane material. At the same time, the diversity of phospholipid biosynthetic pathways across bacterial lineages demonstrates that cells have evolved different biochemical solutions to achieve production and incorporation of membrane lipids. Importantly, the properties of a membrane emerge from the interplay between its chemical composition and its physical organization. Changes in acyl chain saturation, length, and branching, as well as variation in lipid headgroups and their relative abundance, can alter membrane fluidity, curvature, electrostatic interactions, and protein organization. These properties, in turn, influence the activity and localization of membrane‐associated enzymes and protein complexes. Therefore, membrane biogenesis should be viewed in relation between metabolism and membrane biophysics, in which metabolic pathways determine membrane compositions, while the resulting membrane environment provides feedback on the organization and activity of membrane‐associated processes, in which also membrane remodeling plays a role.

A further layer of complexity arises after lipid synthesis. Newly synthesized phospholipids must be distributed between membrane leaflets and, in diderm bacteria, transported between the cytoplasmic and outer membranes. The emerging evidence for dynamic phospholipid asymmetry and dedicated lipid transport systems indicates that membrane expansion is not simply a matter of increasing the total amount of lipid. Instead, cells must control where individual lipid species are deposited and how they are distributed within the cell envelope. The mechanisms governing phospholipid translocation as well as the relationship between lipid transport and membrane growth are still largely unexplored, although research in this field is recently emerging.

Several fundamental questions therefore remain open. To what extent do individual biosynthetic enzymes form functional interaction networks that channel metabolic flux directly toward membrane expansion? How are lipid composition and membrane physical properties sensed and translated into changes in biosynthetic activity? Furthermore, how are newly synthesized lipids spatially distributed within the cytoplasmic membrane and how is this distribution coordinated with cell shape, membrane curvature, and the assembly of division machinery? Taken together, membrane biogenesis is best understood as an integrated process in which biochemical reactions, metabolic flux, lipid chemistry, membrane physics, and cellular organization are intrinsically interconnected. Understanding how these layers are coordinated will be essential for explaining how bacteria maintain membrane homeostasis while simultaneously expanding, adapting to environmental changes, and ultimately dividing.

## Author Contributions

**Sergiy Gan** **:** conceptualization (equal); investigation (lead); project administration (equal); visualization (lead); writing – original draft (lead). **Marten Exterkate** **:** conceptualization (equal); funding acquisition (lead); project administration (equal); writing – review and editing (lead).

## Funding

This study was supported by Federation of European Biochemical Societies Booster Fund.

## Conflicts of Interest

The authors declare no conflicts of interest.

## Supporting information

Supplementary Material

## Data Availability

Data sharing is not applicable to this article as no datasets were generated or analysed during the current study.
