# Supplementary material for: Cytoplasmic Lipid Membrane Biogenesis in Prokaryotes: From Soluble Lipid Building Blocks to a Dynamic and Functional Boundary Layer
Source: Chembiochem. 2026 Oct 23;27(20):e70579. doi: 10.1002/cbic.70579 (PMC13691511; doi:10.1002/cbic.70579)
Supplement: Supplementary file 1 [file CBIC-27-e70579-s001.pdf]

Supplementary

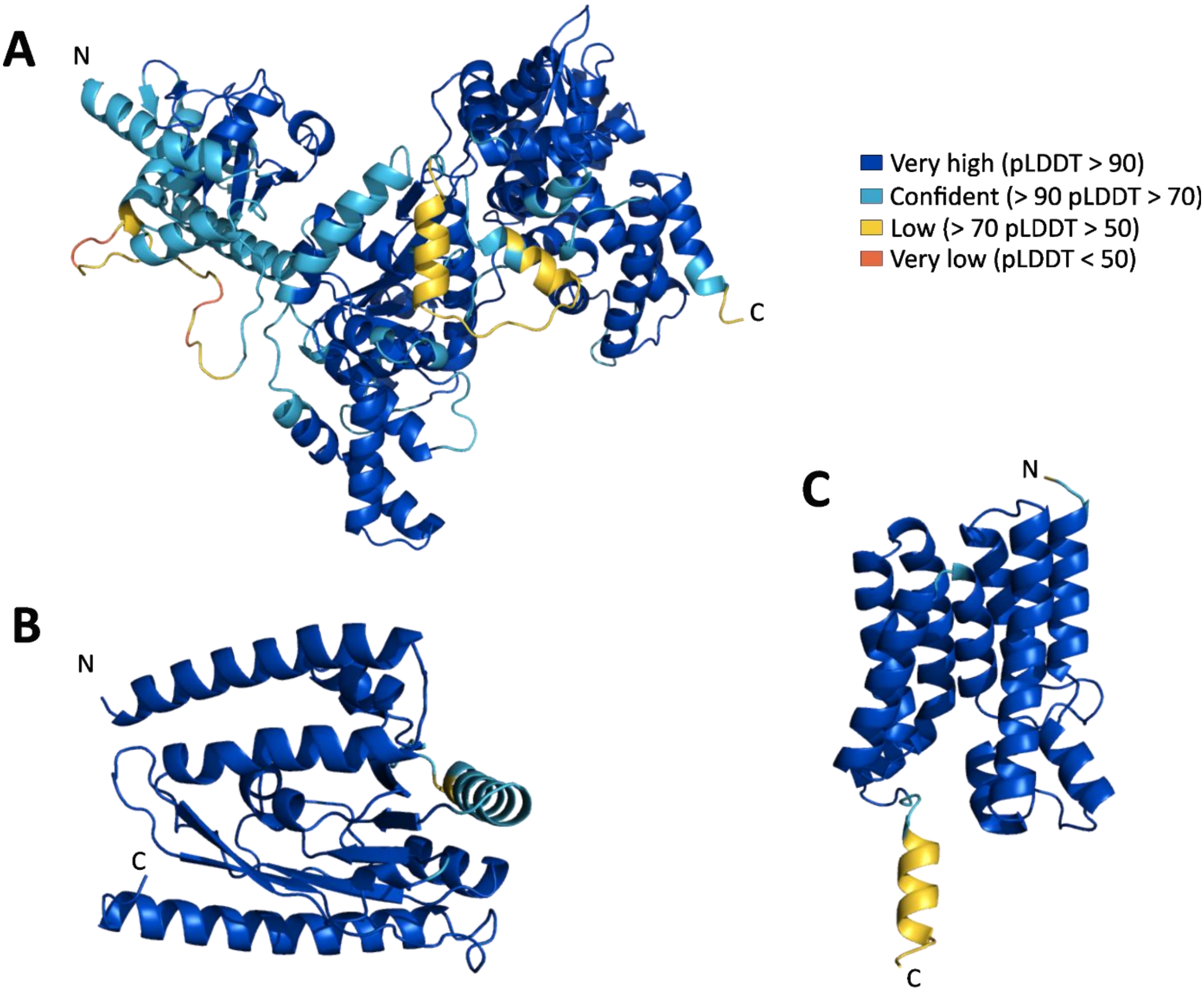

Figure S1*:* Colored AlphaFold 3 model of PlsB (A), PlsC (B) and PlsY (C) from *Escherichia Coli* K12 according to their per residue Predicted Local Distance Difference Test scores (pLDDTs) [91]. The coloring is based on the values stored in the B-factor column of the model PDB file and follows the color scheme used in the AlphaFold EBI protein structure database.
